# Prevalence of Obstetric Danger Signs during Pregnancy and Associated Factors among Mothers in Shashemene Rural District, South Ethiopia

**DOI:** 10.1155/2020/6153146

**Published:** 2020-09-26

**Authors:** Nega Terefe, Aderajew Nigussie, Afework Tadele

**Affiliations:** ^1^District Health Office, Shashemene, Oromia, Ethiopia; ^2^Population and Family Health, Jimma University, Jimma, Ethiopia

## Abstract

**Introduction:**

Obstetric danger signs are those signs that a pregnant woman will see or those symptoms that she will feel which indicate that something is going wrong with her or with the pregnancy. Evidence on the prevalence of obstetric danger signs and contributing factors were crucial in designing programs in the global target of reducing maternal morbidity and mortality.

**Objective:**

To assess the prevalence of obstetric danger signs during pregnancy and associated factors among mothers in a Shashemene rural district, South Ethiopia.

**Methods:**

A community-based cross-sectional study design was conducted among 395 randomly selected women who gave birth in the last six months. A pretested interviewer-administered questionnaire was utilized. Data were cleaned, coded, and entered into Epi data manager version 4.1 and then exported to SPSS version 20. Bivariable and multivariable logistic regression analyses were employed to assess the association between independent variables with the outcome variable. Statistical significance was declared at *p* < 0.05.

**Result:**

One hundred sixty-three (41.3%) of women had a history of obstetric danger signs during pregnancy. The most prevalent obstetric danger signs were vaginal bleeding (15.4%) followed by swelling of the body 12.7% and severe vomiting 5.3%. Women who have less than four times antenatal care visits were 6.7 times more likely to experience obstetric danger signs (AOR 6.7 (95% CI 3.05, 14.85)) compared to those who had antenatal care visit four times and above. Women who have inadequate knowledge of obstetric danger signs were 2.5 times more likely to experience obstetric danger signs during pregnancy (AOR 2.5 (95% CI 1.34, 4.71)), and primigravida women were 6.3 times more likely to have obstetric danger signs during pregnancy (AOR 6.3 (95% CI 2.61, 15.09)) compared to multiparous women.

**Conclusion:**

About half of the pregnant mothers have experienced at least one obstetric danger signs. Public health interventions on maternal health should give priority to the prevalent causes of obstetric danger signs, strengthening completion of four antenatal care visits and health education on obstetric danger signs for pregnant mothers at community level especially for primgravid women.

## 1. Introduction

Pregnancy is a normal process that results in a series of physiological and psychological changes in a pregnant woman. As a result, even normal pregnancy may end in potentially life-threatening maternal and fetal complications. Danger signs during pregnancy are any signs that a pregnant woman will experience or any symptoms that she will feel, which endangers her pregnancy. They are known as obstetric danger signs (ODS), for instance, loss of consciousness; persistent vomiting; severe persistent abdominal pain; vaginal bleeding; swelling of face, fingers, and feet; blurring of vision; fits of pregnancy; severe recurrent frontal headache; and high-grade fever. [1, 2].

Every pregnant woman faces the risk of sudden, unpredictable complications that could end up with death or injury to herself or to her infant that are related to ODS and cannot be reliably predicted [3, 4]. Pregnancy is a crucial time to promote healthy behaviors and parenting skills. Inadequate care during this time breaks a critical link in the continuum of care and affects both women and babies [2]. Consequently, ODS can cause obstetric complications, i.e., growth-restricted fetuses, oligohydramnios, and premature rupture of membranes, preterm labor, and increased rates of cesarean section [4, 5].

Maternal mortality is unacceptably high. According to the World Health Organization (WHO) estimates in 2017, about 295,000 women died due to pregnancy-related complications. The vast majority of these deaths (94%) occurred in low-resource settings, and most could have been prevented. Sub-Saharan Africa alone accounted for roughly two-thirds (196,000) of maternal deaths [6]. According to Ethiopian Demographic Health Survey (EDHS) in 2016, maternal mortality and morbidity in Ethiopia was 412 per 100,000 live births and 30% of these deaths are related to ODS during pregnancy [7].

Many pregnant women and their families in developing countries including Ethiopia have limited understanding of pregnancy danger signs and thereby for which they delay in reaching health facilities even when ODS occurs [1, 3]. Increasing knowledge of pregnancy danger signs is considered a strategy which encourages the utilization of skilled care during pregnancy. ODS could be prevented significantly when a woman and her families recognize ODS as a life-threatening condition and seek health care early [1, 8].

The previous work has only focused on assessing the prevalence of some common ODS separately, for instance, a study in southern Ethiopia found antepartum hemorrhage complicates 20%, hyperemesis complicates 4.8%, and eclampsia occurs around 12% during pregnancy [6] and a study conducted in Arab women showed that the prevalence of vaginal bleeding among Qatar woman was 15.3% and is associated with education level and family history of hypertension [9]; and other studies conducted in Ilu Ababora, Ethiopia, showed that vaginal bleeding 21.6%, severe headache 19.9%, swelling of the hand and face 11%, gush blood from vagina 8.7%, and conversion 5.1% happens during pregnancy [8].

In addition, research has tended to focus on knowledge and practices [10–13] of ODS rather than the prevalence of ODS. However, this study was conducted on rural women to determine the overall prevalence of ODS including women who did not attend maternal health services on the primary prevention of direct causes of maternal mortality. Evidence-based early intervention of ODS during pregnancy has a substantial impact on health, social, economic, political, and environmental issues. Therefore, this study is aimed at determining the prevalence of ODS and contributing factors in Shashemene rural district, Southern Ethiopia.

## 2. Materials and Methods

### 2.1. Study Area and Period

The study was conducted in the Shashemene district, located in West Arsi Zone, Oromia regional state, Ethiopia. The total population of the district was estimated to be 265,109 based on the woreda health office report of 2018. The district was 225 km south of Addis Ababa, the capital city of Ethiopia. It was divided into 33 kebeles (small administrative units) with health infrastructures of 8 health centers and 33 health posts. The study was conducted from April 20 to May 21, 2018.

### 2.2. Study Design

The study has a community-based cross-sectional study design.

### 2.3. Study Population

Randomly selected mothers who gave birth in the Shashemene rural district within the last six months during the data collection period were included, while women who are seriously ill and unable to perform interviews and mothers who complain illness of their neonates and/or infants were excluded.

### 2.4. Sample Size Determination

The sample size of the study was determined by using the Epi Info version 7.1.1 StatCalc with the assumptions of 95% confidence level, *p* = prevalence of mothers experienced vaginal bleeding was 19.1% (10), *d* =4% (marginal errors), Finally, by adding a nonresponse rate of 10%, *n* = 407.

### 2.5. Sampling Techniques

Ten kebeles were selected by using a simple random sampling (lottery) method from thirty-three kebeles in the Shashemene rural district. Then, the census was conducted to register all mothers who gave birth within the last six months to prepare the sampling frame. Proportion to size allocation for each of the ten kebeles based on the number of eligible mothers for the study was done based on census results. Code given for households of eligible mothers during the census was used as a sampling frame for the final selection of the mothers. Finally, computer-generated random numbers were used to recruit study participants.

### 2.6. Operational Definitions

*Obstetric danger signs* (ODS) refers to the loss of consciousness; persistent vomiting; severe persistent abdominal pain; vaginal bleeding; swelling of face, fingers and feet; blurring of vision; fits of pregnancy; severe recurrent frontal headache; and high-grade fever.

*Gravidity* refers to a total number of pregnancies.

*Kebele* is the lowest administrative structure next to the district.

### 2.7. Measurement

#### 2.7.1. ODS

A woman who experienced at least one of the ODS (loss of consciousness; persistent vomiting; severe persistent abdominal pain; vaginal bleeding; swelling of face, fingers, and feet; blurring of vision; fits of pregnancy; severe recurrent frontal headache; and high-grade fever) was categorized as has ODS and no ODS otherwise.

#### 2.7.2. Knowledge of ODS

Knowledge of ODS was assessed by asking 21 questions, and participants who scored a mean above score was categorized as having adequate knowledge of ODS, otherwise inadequate knowledge [10–13].

### 2.8. Survey Administration

The questionnaire was prepared after a review of different literature and modified to suit and relate to the study objective and the area's context from different materials. Questionnaires have sociodemographic factor, maternal factor, and health facility-related factor parts. The questionnaire was partially adapted from the survey tools developed by JHPIEGO Maternal and Neonatal Health program and contextualized according to local contexts. The questionnaire was adapted to fit the study area population context and to meet the objectives of the study. An interviewer-administered structured questionnaire was used to collect the data from mothers who gave birth in the Shashemene district. The questionnaires were first developed in English and then translated into Afaan Oromo, and then translated back to English again to check its consistency.

Six diploma nurses with experiences in survey data collection and two health officers as supervisors participated in the data collection process after two-day training was given by the principal investigator. During the data collection period, the data collectors and supervisors were guided by health development army leaders in each kebele so that they can easily access the houses of each sampled house of women who gave birth within the last six months. The data collectors were given the list of women who gave birth within the last six months in each kebele to be interviewed. The pretest was carried out at Arsi Nagelle district, five days before the actual data collection date, which was outside of the study area and has similar sociodemographic characteristics.

During the procedure, the data collectors interview the participants in a private area to increase the confidentiality of the participants.

#### 2.8.1. Data Management and Quality

Various activities were performed to assure the quality of data, and data collectors were selected carefully based on clearly established criteria of diploma nurses who were experienced in data collections and currently not working in the kebeles.

Before data collection, both interviewers and supervisors were trained in the interview approach, ways to maintain confidentiality, and the privacy of the study participants for two days. The appropriateness of the questionnaire in terms of content, consistency, language, and organization was checked and was modified.

The English version prepared questionnaire was translated to the local language (Afaan Oromo) by a person knowing both the languages. Then, another individual who had very good knowledge of both English and Afaan Oromo language translated the Afaan Oromo version back to English to check for its original meaning.

The questionnaire was pretested on 21 respondents (5%of sample size) in Arsi Nagelle woreda that had similar characteristics with the study population. The pretest findings were discussed among data collectors, supervisors, and the investigator to ensure a better understanding of the data collection process. Based on the pretest, questions were revised, edited, and those found to be unclear or confusing were modified. To reduce nonresponse rate and unwanted confusion, necessary information and description were given to respondents before initiating the interview. Finally, a structured Afaan Oromo version questionnaire was used for data collection. The principal investigator and supervisor supervised the data collection process. The data quality was controlled by close supervision with aggressive monitoring. Every day, 10% of the completed questionnaires were reviewed and checked for completeness and consistency by the supervisors and principal investigator and the necessary feedback offered to data collectors in the next morning before the data collection begins.

#### 2.8.2. Data Processing and Analysis

To control the quality of the data processing, the data was checked for its completeness before data entry and the inconsistent data was checked to refer to the hard copy of the questionnaire. Quantitative data were entered into Epi data manager version 4.1 and exported to SPSS version 20 for analysis. The cleaning process was done by running a simple frequency after data entry for its consistency. Errors related to inconsistency of data such as missing values and outliers were checked and considered during data cleaning.

Descriptive statistics using frequencies, percentages, mean, and standard deviation were used to describe findings. The frequency distributions of the variables were worked out using tables and figures.

Bivariable analysis using logistic regression was done and all explanatory variables which have an association with the outcome variable at a *p* value of less than 0.25 were selected as candidates for multivariable analysis. Multicollinearity between the candidate variables was checked with a minimum tolerance level at 0.2.

Hence, variables with a *p* value of less than 0.25 in the bivariate logistic regression analysis were entered into a multivariable logistic regression model.

Then, multivariable analysis using a backward stepwise selection method was done to control for possible confounding variables and to determine the presence of a statistically significant association between explanatory variables and the outcome variable.

The level of statistical significance was declared at a *p* value of < 0.05, and AOR with 95% CI was used to measure the degree of association between independent variables and the outcome variable. Model fitness was checked using Hosmer and Lame show goodness-of-fit test. Finally, the dependent variable was organized as a binary variable with two categories: ODS present (1) and absent (0).

The principal component analysis was conducted to set the wealth/economic status of pregnant women. Before analysis, sample adequacy was checked, and after, the assumption of sampling adequacy was fulfilled; then, the appropriateness of the principal component analysis was checked. After that, variables were included and removed where decided. Then, principal component extraction was used to extract variables. The correlation coefficient between the variables (rows) and the principal component was checked. A total of 9 items on household assets were analyzed using the principal component analysis method after checking the fulfillment of assumptions using the Kaiser-Meyer-Olkin measure of sampling adequacy and Bartlett's test of sphericity. Finally, the wealth status of pregnant mothers was classified as low wealth status, medium-high wealth status, and high wealth status depending on the mean value of assets of the mother's score.

## 3. Result

### 3.1. Sociodemographic Characteristics of Participants

In this study, a total of 395 women who gave birth in the last six (6) months were interviewed from the randomly selected kebeles in the Shashemene district comprising a response rate of 97%.

Among a total of 395 participants, most of them 127 (32.7%) were found between age 20 and 24 years with the mean age (standard deviation (SD)) of 27.84 (±6.219) and about more than half (53.9%) of the women had their first pregnancy after the age of 18 years, with mean (SD) age at first pregnancy 19.16 (±2.651). Only fourteen (3.5%) participants were not in a marital union (Table 1).

### 3.2. Obstetric Characteristics of Mothers Who Gave Birth in Less than Six Months in Shashemene District, Southern Ethiopia, 2018

The majority of the women (83.5%) were multiparous. More than half of the pregnancies (87.3%) were planned, while the remaining 12.7% were unplanned pregnancy. Two hundred and two women visited antenatal care (51.1%) less than four times (Table 2).

### 3.3. Prevalence of Obstetric Danger Signs during Pregnancy, in Shashemene District, 2018

**Figure 1:**
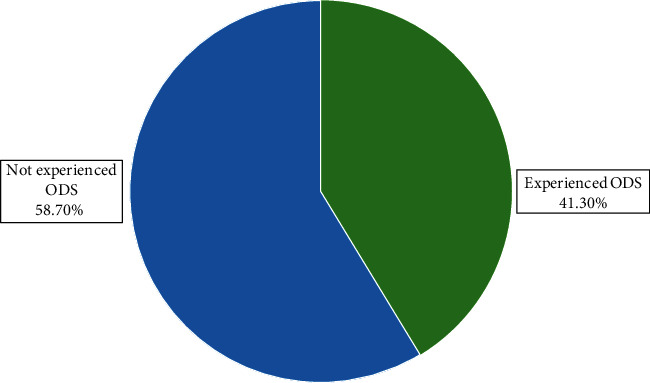
Prevalence of obstetric danger signs during pregnancy in Shashemene district, 2018.

**Figure 2:**
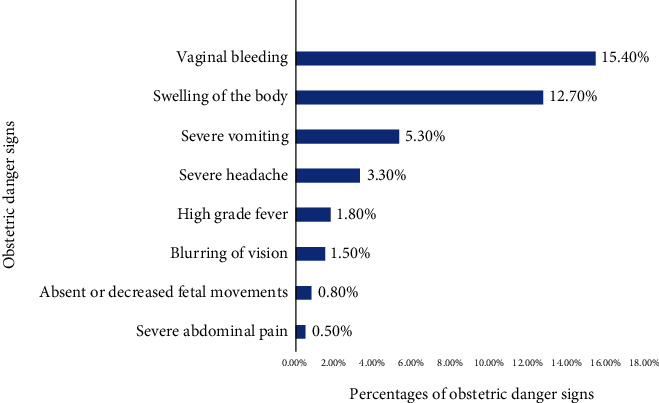
Types of obstetric danger signs during pregnancy in Shashemene district, 2018.

Two hundred and thirty-two (58.7%) of the study participants had no history of ODS during pregnancy (Figure 1). The most prevalent ODS was vaginal bleeding 15.4%, followed by swelling of the body (12.7%) and severe vomiting (5.3%) (Figure 2).

### 3.4. Factors Associated with ODS during Pregnancy among Women Who Gave Birth in the Last Six Months in the Shashemene District

Maternal antenatal care follow-up was found to be a significant predictor of experiencing ODS. Mothers who visit antenatal care less than four times were 6.7 times (AOR 6.7 (95% CI 3.05, 14.85)) more likely to experience ODS during pregnancy as compared to their counterparts. Mothers who were less knowledgeable about ODS were 2.5 times more likely to experience ODS (AOR 2.5 (95% CI 1.34, 4.71)), and those who gave birth once were 6.3 times more likely to experience ODS (AOR 6.3 (95% CI 2.61, 15.09)) compared to multiparous women (Table 3).

## 4. Discussion

Maternal death was unacceptably high especially in rural settings where maternal health services were low and absent. Early identification and management of ODS help in the prevention of maternal mortality in these settings globally. This community-based study identified the prevalence and factors that influence the ODS during pregnancy among mothers who gave birth in the past six months before this survey in the Shashemene rural district in Southern Ethiopia.

The study found 41.3% overall prevalence of ODS in the area. This finding was higher than the study conducted in Egypt and Nigeria [7, 14] with 4.5% and 29.6%, respectively. This difference may be the use of different study areas; studies in Egypt and Nigeria were facility-based study, and still, not all maternal health service coverage was not 100%. On the other hand, this finding was lower than a study conducted in South Africa and Egypt [12, 15], with the prevalence of ODS in those studies being 79.7% and 62.5%, respectively. These differences may be differences in the study period.

The most prevalent ODS during pregnancy in the study area was vaginal bleeding followed by swelling of the body and severe vomiting. This finding was similar to the study conducted in Egypt and Ilu Ababora, Ethiopia [15, 16]. This might imply that still ODS is prevalent in Ethiopia.

There was a significant positive association between ODS and antenatal care follow-up. Women who visited a health facility for pregnancy less than four times were more than six times more likely to experience an ODS during pregnancy as compared to their counterparts. This is in good agreement with the standard WHO recommendation of antenatal care follow-ups and other findings [4, 13, 14]. This similarity may be mothers that visited ANC get adequate health information related to ODS during pregnancy.

Knowledge of ODS showed a strong statistical association with the experience of ODS during pregnancy. Those who have inadequate knowledge of ODS were about more than twice more likely to experience ODS during pregnancy. This lends support to a study conducted in Egypt, and Ilu Ababora (Ethiopia) [7, 10]. The association between ODS and knowledge of ODS is worth mentioning because identifying warning signs of ODS would make women seek health care early before experiencing the ODS. When a woman knows every pregnancy as risky and gets knowledge of ODS, she will take care of herself thereby preventing ODS.

Finally, the number of pregnancies was significantly associated with ODS during pregnancy. Being a primigravida mother was more than six times at higher odds of experiencing ODS than multigravida mothers. This substantiates previous findings in the literature [8, 17]. The possible explanation for this result is that most of the ODS was related to the physiological and psychological conditions of the women, as it was higher in the primigravid women. Women who had experience of pregnancy may predict what they experience throughout the pregnancy and tends to seek care early before the actual experience of the ODS.

Being a community-based study can show the problem well as maternal health services in the region were still very low. However, it is plausible that several limitations could have influenced the results obtained. To begin with, the design being cross-sectional makes it difficult to establish the cause-effect relationships of the outcome variable with the independent predictors. Additionally, recall bias may affect the actual response of the study participants.

## 5. Conclusion

About one in two women experienced at least one ODS in the study area. Public health interventions on maternal health should give priority to the prevalent causes of obstetric danger signs, i.e.. vaginal bleeding, swelling of the body, and severe vomiting, strengthening completion of four antenatal care visits and health education on obstetric danger signs for pregnant mothers at the community level especially for primigravid women at the community-based level. The health workers and health extension workers should create awareness on ODS. Further studies were recommended to explore the sociocultural predictors of ODS and related practices of the rural community and a longitudinal study to determine the causes of the most prevalent ODS.

## Figures and Tables

**Table 1:** Sociodemographic characteristics of mothers who had less than a six-month child and their husbands in Shashemene district, Southern Ethiopia, May 2018.

Variables	Category	Frequency	Percent (%)
Ethnicity	Oromo	195	49.4
	Amhara	113	28.6
	Kambata	47	11.9
	Wolayita	28	7.1
	Others^a^	12	3
Age	15-19	112	28.4
	20-24	127	32.2
	25-29	82	20.7
	30-34	41	10.3
	≥35	33	8.4
Marital	In marital union	381	96.5
	Not in marital union^b^	14	3.5
Religion	Protestant	188	47.6
	Muslim	146	37
	Orthodox	41	10.4
	Others^c^	20	5
Respondents education	No formal educations	39	9.9
	Primary educations	180	45.6
	Secondary and above educations	176	44.6

Others: ^a^Gurage, Tigre; ^b^widowed, divorced, or separated; ^c^wakefata and catholic.

**Table 2:** Obstetric characteristics of mothers who had less than a six-month child in Shashemene district, Southern Ethiopia, May, 2018.

Variables	Category	Frequency	Percent (%)
ANC visits	Yes	270	68.4
	No	125	31.6
Number of ANC visit	<4	202	74.8
	≥4	68	25.2
Planned pregnancy	Yes	50	12.7
	No	345	87.3
Gravidity	One	65	16.5
	Two and above	330	83.5
Multiple pregnancies	Yes	6	1.5
	No	389	98.5
Accessibility of transports	Yes	225	57
	No	170	43
Counseled on obstetric danger sign at ANC	Yes	207	52.4
	No	63	47.6

**Table 3:** Multivariable logistic regression results and factors significantly associated with obstetric danger signs during pregnancy, in Shashemene district, 2018.

Variables	Category	Experienced obstetric danger signs		COR (95% CI)	AOR (95% CI)
		Yes	No		
Husband educational status	No formal educations	61	30	2.56 (1.46,4.503)	1.42 (0.63,3.24)
	Primary educations	117	65	2.27 (1.42,3.62)	1.73 (0.85,3.48)
	Secondary and above	54	68	1.0	1.0
Number of ANC visit	Less than four	144	58	11.59 (5.79,23.19)	6.7 (3.1,14.85)^∗∗^
	Greater or equal to four	12	56	1.0	1.0
Attitude	Good attitude	92	97	1.0	1.0
	Poor attitude	71	135	1.8 (1.21,2.70)	0.58 (0.28,1.2)
Knowledge	Inadequate knowledge	169	55	5.27 (3.41,8.14)	2.51 (1.34,4.71)^∗^
	Adequate knowledge	63	108	1.0	1.0
Gravidity	One times	152	11	4.19 (2.12,8.31)	6.3 (2.6,15.1)^∗∗^
	Two and above	178	54	1.0	1.0

^∗∗^ represents *p* value of less than 0.001 and ^∗^*p* value < 0.05.

## Data Availability

The data sets used and/or analyzed during the current study are available from the corresponding author on reasonable request.

## Abbreviations

ANC: Antenatal care
CHW: Community health workers
EDHS: Ethiopian Demographic Health Survey
SPSS: Statistical Package for Social Science
WDA: Women development army
WHO: World Health Organization.
